# Role of long non-coding RNAs in metabolic reprogramming of gastrointestinal cancer cells

**DOI:** 10.1186/s12935-023-03194-0

**Published:** 2024-01-06

**Authors:** Kang Wang, Yan Lu, Haibin Li, Jun Zhang, Yongle Ju, Manzhao Ouyang

**Affiliations:** 1 Department of Gastrointestinal Surgery, Shunde Hospital, Southern Medical University, The First People’s Hospital of Shunde Foshan), Shunde, Foshan, 528300 Guangdong China; 2 The Second School of Clinical Medicine, Southern Medical University, Guangzhou, 510080 Guangdong China; 3 Guangdong Medical University, Dongguan, 523808 China

**Keywords:** Long non-coding RNAs, Gastrointestinal tract tumors, Metabolic reprogramming, Metabolic enzymes, microRNA, Tumor microenvironment, Prognosis, Therapeutic targets

## Abstract

Metabolic reprogramming, which is recognized as a hallmark of cancer, refers to the phenomenon by which cancer cells change their metabolism to support their increased biosynthetic demands. Tumor cells undergo substantial alterations in metabolic pathways, such as glycolysis, oxidative phosphorylation, pentose phosphate pathway, tricarboxylic acid cycle, fatty acid metabolism, and amino acid metabolism. Latest studies have revealed that long non-coding RNAs (lncRNAs), a group of non-coding RNAs over 200 nucleotides long, mediate metabolic reprogramming in tumor cells by regulating the transcription, translation and post-translational modification of metabolic-related signaling pathways and metabolism-related enzymes through transcriptional, translational, and post-translational modifications of genes. In addition, lncRNAs are closely related to the tumor microenvironment, and they directly or indirectly affect the proliferation and migration of tumor cells, drug resistance and other processes. Here, we review the mechanisms of lncRNA-mediated regulation of glucose, lipid, amino acid metabolism and tumor immunity in gastrointestinal tumors, aiming to provide more information on effective therapeutic targets and drug molecules for gastrointestinal tumors.

**Graphical Abstract:**

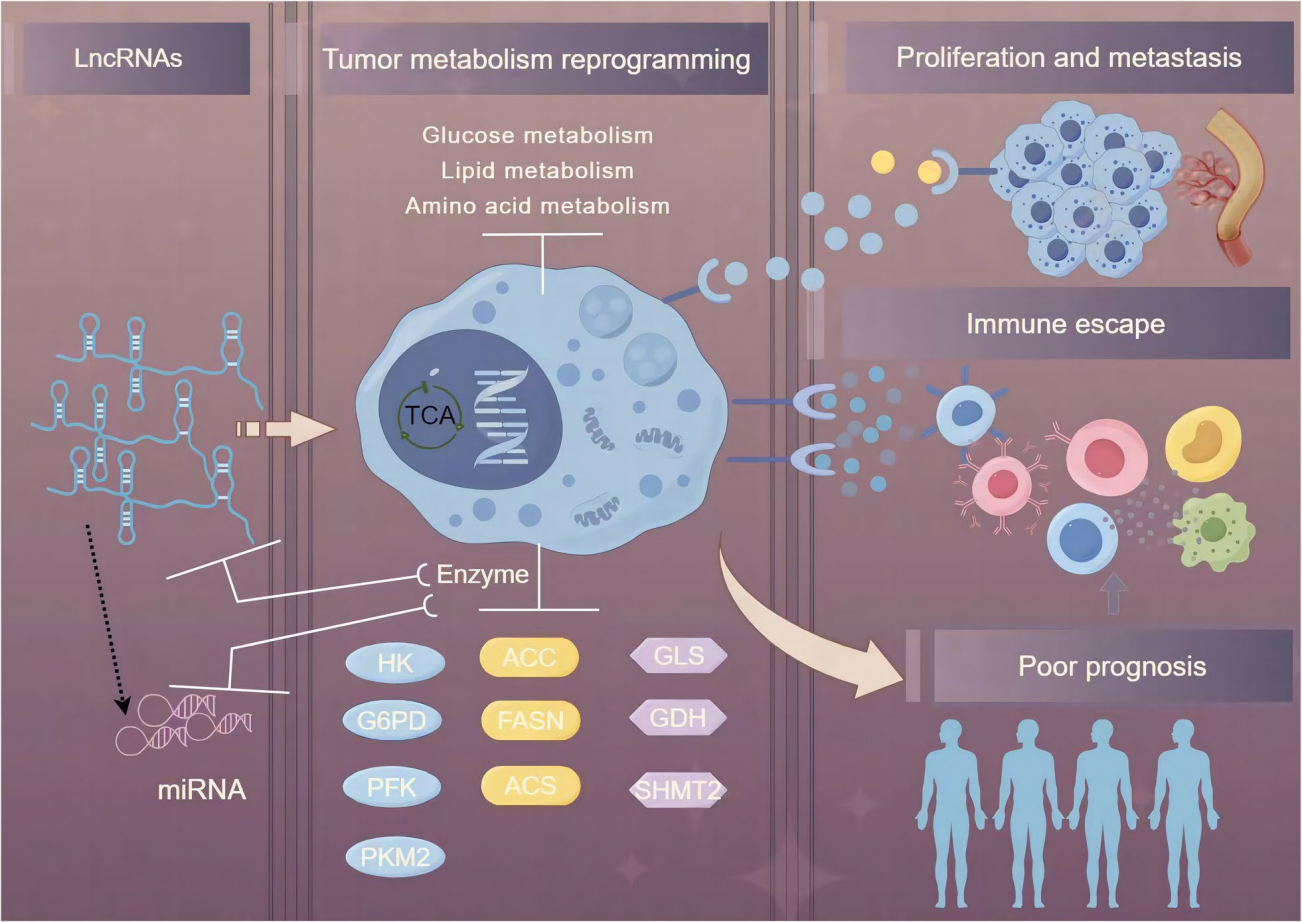

## Introduction

The incidence of gastrointestinal (GI) tract tumors is on the rise globally due to the effect of dietary patterns, obesity, and lifestyle factors [1]. GI cancer is the most commonly diagnosed cancer and is the main cause of cancer-related deaths worldwide. The four most common types of GI tract tumors, namely colon, gastric, liver, and esophageal cancers (EC), present a high mortality rate due to factors such as metastasis, late diagnosis, and postoperative recurrence. Although the incidence of pancreatic and gallbladder cancers is not higher than that of the above cancer types, their hidden and atypical clinical symptoms lead to a low rate of early diagnosis and a very poor prognosis. Although many other tumors in the digestive system have been identified, few studies have explored the effect of lncRNAs on tumor development. Therefore, this review details the mechanisms of action by which lncRNAs are involved in the metabolic reprogramming of GI tract tumor cells.

**Fig. 1:**
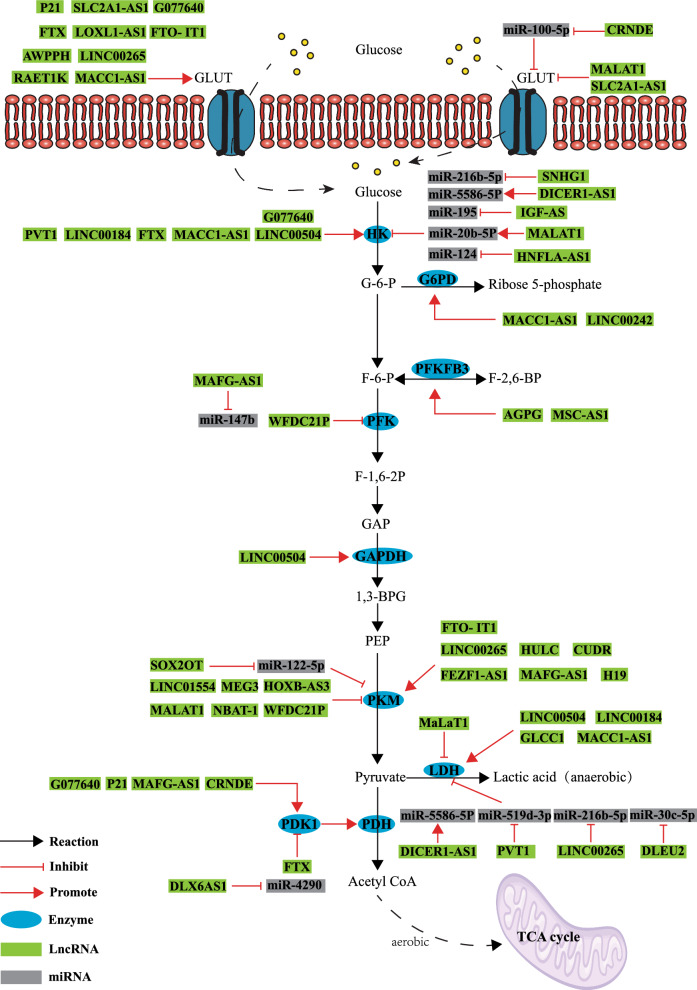
LncRNAs participate in glucose metabolism reprogramming of gastrointestinal cancer cells

**Fig. 2:**
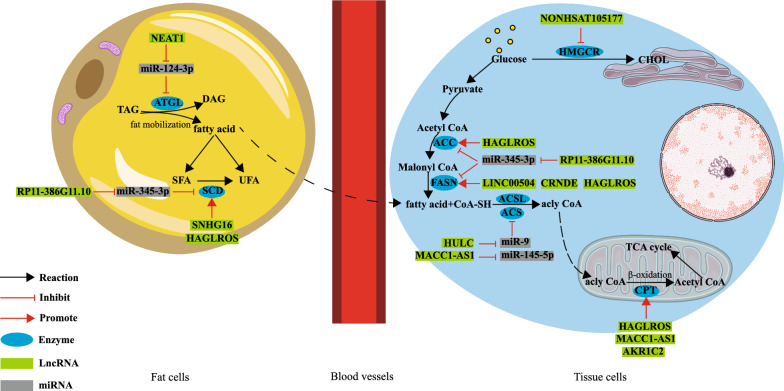
LncRNAs affect lipid metabolism reprogramming in tumors of gastrointestinal cancer cells

**Fig. 3:**
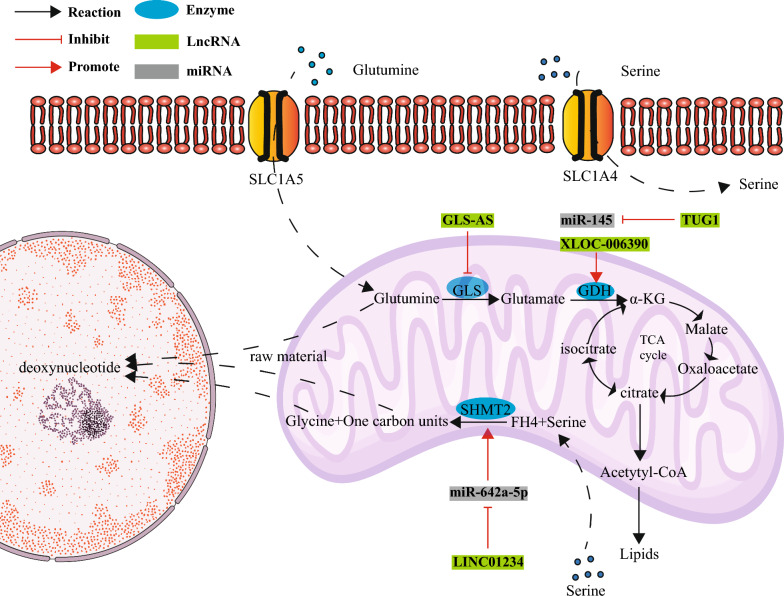
LncRNAs involved in amino acids metabolic reprogramming of gastrointestinal cancer cell

The onset of metabolic changes is a salient feature of tumor cells. Compared with normal cells, the metabolic changes in tumor cells are the result of metabolic reprogramming, which endows cancer cells with strong survival and proliferation ability [2]. Moreover, metabolic reprogramming renders the tumor cells resistant to chemotherapy and radiation therapy [3]. As the first metabolic change in tumor cells, the Warburg effect facilitates tumor cells to obtain energy through increased glycolysis [4]. Normal cells obtain energy by oxidizing sugar molecules through oxidative phosphorylation, while most tumor cells, including those of GI tract tumors, obtain energy through glycolysis [5]. The rate of glycolysis in malignant, fast-growing tumor cells is up to 200 times higher than that of normal tissues. Because the rate of glycolysis is enhanced even in the presence of oxygen, the Warburg effect is also referred to as aerobic glycolysis [6]. Research has revealed that tumor cells undergo many metabolic changes. In GI tract tumors, in addition to glycolysis and oxidative phosphorylation, metabolic reprogramming affects the pentose phosphate pathway, tricarboxylic acid cycle, lipid metabolism, and amino acid metabolism to meet the growth of tumor cells (Figs. 1–3) [7–9]. The enhanced rate of pentose phosphate pathway and fatty acid (FA) metabolism leads to the accumulation of phospholipids, cholesterol, and ribose-5 phosphoric acids, which serve as the raw materials for cell membrane formation and nucleotide components for tumor cell division [10, 11]. At the same time, the enhanced rate of glycolysis leads to a reduction in the rate of the citric acid cycle. To compensate for the deficiency of the citric acid cycle, cancer cells exhibit upregulated glutamine metabolism. Glutamine is used to maintain the potential and integrity of the mitochondrial membrane and to promote the production of NADPH, which is needed for macromolecular synthesis and redox control, thereby promoting tumor proliferation. Glutamine addiction is a major characteristic associated with the changes in amino acid metabolism in tumor cells [12].

LncRNAs, or long-chain non-coding RNA molecules, are greater than 200 nucleotides in length and lack protein-coding capabilities. They exert crucial regulatory functions within cells. Along with the development of high-throughput sequencing technology, more and more lncRNAs have been discovered [13]. LncRNAs are usually transcribed by RNA polymerase II, and there is no clear and uniform standard for their classification. LncRNAs are often classified according to their transcriptional location in the genome, which determines their mechanism of action and functions [13–18]: (1) Intergenic lincRNAs (lincRNAs): These lncRNAs are transcribed from DNA sequences located between two protein-coding genes; (2) Sense lncRNAs: These lncRNAs are transcribed in the same direction as the adjacent protein-coding genes; (3) Antisense lncRNAs: These lncRNAs are transcribed in the opposite direction to the protein-coding genes; (4) Bidirectional lncRNAs: These lncRNAs share the same promoter as neighboring protein-coding genes but are transcribed in the opposite direction; (5) Intronic RNAs: These lncRNAs are transcribed from introns, which are non-coding regions within protein-coding genes; (6) Enhancer-associated lncRNAs: These lncRNAs mainly originate from enhancer regions associated with protein-coding genes. Based on their functions, lncRNAs can be categorized into four groups: signaling molecules, decoy molecules, guidance molecules, and scaffolding molecules [19]. As signaling molecules, lncRNAs participate in certain signaling pathways and regulate downstream gene transcription; as decoy molecules, lncRNAs can bind to and remove some transcription factors or proteins to regulate gene expression; as guide molecules, lncRNAs bind to protein complexes with regulatory or enzymatic activities and guide them to target to gene promoters or specific genomic sites to regulate downstream signaling events and gene expression; as scaffolding molecules, by binding to different proteins, lncRNAs are capable of forming complexes that can modify histones on chromatin, etc. In a variety of ways, lncRNAs are responsible for regulating gene expression. Acting as scaffolds, lncRNAs can bind to a range of proteins, forming complexes that are capable of modifying histones on chromatin, among other functions. There are a variety of ways in which lncRNAs regulate the expression of genes. LncRNAs do not encode proteins, but they mainly regulate gene expression at the epigenetic, transcriptional, and post-transcriptional levels, thus affecting cell cycle regulation, cell differentiation, metabolic regulation, and many other aspects regulation, metabolic regulation and other biological processes. For example, LncRNA INHEG is highly expressed in glioma stem cells (GSCs), enhances the interaction between NOP58 and TAF15, increases the sumoylation of NOP58, and participates in the biogenesis of the C/D box snoRNP process, as well as increases the methylation of the ribosomal RNA 2ʹ -O, and the ratio of the ribosomal RNA sites along the ribosomal RNA locus at the post-transcriptional level, which promotes the translation of the mRNAs, and maintains self-renewal of the GSCs [20]. LINC00689 acts as a ceRNA to promote PKM2 expression in glioma cells by sponging miR-338-3p, which promotes glioma cell proliferation, migration, invasion, and glycolysis [21]. The activity and synthesis of metabolic enzymes play a key role in the metabolic reprogramming of tumor cells [22]. Although lncRNAs do not encode proteins, they affect the metabolism of tumor cells and regulate the expression of various metabolic enzymes at both mRNA and protein levels. LncRNAs can directly bind to the promoter of genes encoding rate-limiting enzymes and regulate their transcription (Table 1–3) [23]. Although the research on lncRNAs has developed rapidly in recent years, there are still a lot of basic and applied problems that need to be solved. In this review, we highlight the role of lncRNAs in different metabolic pathways of GI tract tumors and explore the mechanism by which lncRNAs regulate metabolism in GI tract tumors, to provide more effective targets and a more reliable theoretical basis for the treatment of GI tract tumors.

**Table 1:** LncRNAs involved in the regulation of glucose metabolism of gastrointestinal cancer cells

Tumor type	LncRNA	Expression	Metabolism-related enzyme	Metabolic process	Clinical prognosis	Ref
Colon cancer	LINC00504	Up	LDHA, HK2, GADPH	Glucose uptake, glycolysis, pentose phosphate pathway	–	[39]
	GLCC1	Up	LDHA	Glycolysis	Poor	[40]
	LINRIS	Up	GLUT-1, PKM2, LDHA	Glycolysis, glucose uptake	Poor	[41]
	HOXB-AS3	Down	PKM2	Glycolysis	Poor	[42]
	FEZF1-AS1	Up	PKM2	Glycolysis	Poor	[43]
	MAFG-AS1	Up	PDK1, PFK1, PKM2	Glycolysis, Krebs cycle	Poor	[44]
	HNF1A-AS1	Up	HK2	Glycolysis	Poor	[45]
	CRNDE	Up	GLUT, PDK4	Glucose uptake, glycolysis	–	[46]
	LINC00265	Up	GLUT, PKM, LDH	Glucose uptake, glycolysis	Poor	[47]
	LincRNA-p21	Down	PDK, PDH	Glucose uptake, glycolysis	–	[48]
	MALAT1	Up	GLUT1, HK2, PKM2, LDH	Glycolysis, glucose uptake	–	[49]
	AWPPH	Up	GLUT1	Glucose uptake	–	[50]
	HCG11	Up	PDK4	Glycolysis	Poor	[51]
Gastric cancer	MACC1-AS1	Up	HK2, GLUT1, LDHA, G6PD	Glycolysis, glucose uptake, pentose phosphate pathway	Poor	[52]
	Ftx	Up	HK2	Glycolysis	–	[53]
	DLX6-AS1	Up	PDK1	Glycolysis	Poor	[54]
	IGF2-AS	Up	HK2	Glycolysis, glucose consumption, and lactate production	Poor	[55]
	MSC-AS1	Up	PFKFB3	Glycolysis	Poor	[56]
	LINC00242	Up	G6PD	Glycolysis	Poor	[57]
	SNHG1	Up	HK2	Glycolysis	Poor	[58]
	DLEU2	Up	LDHA	Glycolysis	Poor	[59]
Liver cancer	Ftx	Up	PDK1, GLUT1, GLUT4, PFK, LDH	Glycolysis, glucose uptake	Poor	[60]
	MEG3	Down	PKM2	Glycolysis	Poor	[61]
	CUDR	Up	PKM2	Glycolysis	–	[62]
	LINC01554	Down	PKM2	Glycolysis	Poor	[63]
	H19	Up	PKM2	Glycolysis	–	[64]
	WFDC21P	Down	PFK, PKM2	Glycolysis	Poor	[65]
	RAET1K	Up	LDHA	Glucose uptake	Poor	[66]
	MALAT1	Up	HK2, GLUT4	Glycolysis, glucose uptake	–	[67]
	SOX2OT	Up	PKM2	Glucose uptake, glycolysis lactate production	Poor	[68]
	SLC2A1-AS1	Down	GLUT1	Glycolysis	Poor	[69]
	LOXL1‑AS1	Up	GLUT	Glucose uptake	Poor	[70]
	FTO- IT1	Up	GLUT1, PKM2	Glucose uptake, glycolysis	Poor	[71]
Esophageal cancer	NBAT-1	Down	PKM2	Glycolysis	Poor	[72]
	HOTAIR	Up	HK2	Glycolysis	Poor	[73]
	AGPG	Up	PFKFB3	Glycolysis	Poor	[74]
	LINC00184	Up	HK, LDHA, PDH	Glycolysis	–	[75]
	SLC2A1-AS1	Up	GLUT1	Glycolysis, glucose uptake	Poor	[76]
	G077640	Up	GLUT4, HK2, PDK1	Glycolysis, glucose uptake	Poor	[77]
Pancreatic cancer	MACC1-AS1	Up	PK	Glycolysis	Poor	[78]
	HOTAIR	Up	HK2	Glycolysis, glucose uptake	Poor	[79]
	LINC01448	UP	HK2	Glucose consumption, lactate production	Poor	[80]
	PVT1	Up	HK2, GLUT1, LDHA	Glycolysis, glucose uptake	Poor	[81]
	DICER1-AS1	Down	HK2, LDHA	Glycolysis	Poor	[82]
Gallbladder cancer	PVT1	Up	HK2	Glycolysis	Poor	[83]

*GLUT* Glucose transporter, *HK* Hexokinase, *PK* Pyruvate kinase, *LDH* Lactate dehydrogenase, *PDK* Pyruvate dehydrogenase kinase, *PFK* Phosphofructokinase, *PKM2* Pyruvate kinase type M2, *LDHA* Lactate dehydrogenase A, *GADPH* Glyceraldehyde-3-phosphate dehydrogenase, *G6PD* Glucose-6-phosphate dehydrogenase, *PFKFB3* 6-phosphate fructose-2-kinase, *PDH* pyruvate dehydrogenase

## LncRNAs participate in glucose metabolism in GI tract tumors through metabolic reprogramming

Despite the presence of ample oxygen, tumor cells exhibit a preference for the glycolytic pathway, known as the Warburg effect, to acquire energy for their growth [24]. The Warburg effect has been observed in a variety of tumors, including gastric, colon, liver, esophageal, pancreatic, and gallbladder cancers [25–30]. The enhancement of glycolysis is related to the upregulation of some key enzymes in this pathway, such as hexokinase-2 (HK2) [31], pyruvate kinase type M2 (PKM2) [32], lactate dehydrogenase (LDHA) [33], and 6-phosphate fructose-2-kinase/fructose-2, 6-bisphosphatase 3 (PFKFB3) [34]. The overexpression of crucial glycolytic enzymes in tumor cells results in multiple alterations, including (a) increased ATP production to meet the high energy demands of tumor cells, (b) enhanced glycolytic flux leading to the accumulation and redirection of glycolytic intermediates for the synthesis of cancer biomass, and (c) the accumulation of lactic acid, promoting tumor progression and contributing to tumor acidosis. This acidic microenvironment synergistically supports tumor advancement, and resistance against specific anticancer treatments, and compromises the effectiveness of antitumor immunity [35].

Glucose transporters (GLUTs) play a key role in the transport of glucose from capillaries to cells. Enhanced activity or expression of GLUTs leads to increased glucose uptake in tumors [36]. Through the pentose phosphate pathway, glucose can be metabolized to provide tumor cells with multiple reducing agents, including a significant amount of NADPH molecules. Additionally, the byproduct 5-phosphate ribose (R5P) can serve as an essential building block for tumor genetic material synthesis [37]. Moreover, glucose-6-phosphate dehydrogenase (G6PD) is the key enzyme in the production of R5P. A new study revealed that the activity and expression of G6PD are enhanced in colon cancer (CC) cells and that increased G6PD expression is associated with aggressive CC behavior [38].

Several studies have shown that lncRNAs play a significant role in the reprogramming of glucose metabolism in GI tract tumor cells (Fig. 1 and Table 1). Next, we will explain the role of lncRNAs in regulating the function of key metabolic enzymes in different digestive system tumor cells, including colon, gastric, liver, esophageal, pancreatic, and gallbladder tumor cells.

### LncRNAs participate in glucose metabolism in colon cancer (CC)

LncRNAs have been shown to modulate multiple metabolic processes in CC cells. LncRNAs modulate the activity of key glycolytic enzymes. The protooncogene c-MYC regulates glucose metabolism in cancer cells. The c-MYC-mediated glucose metabolism in CC cells is regulated by several lncRNAs, such as LINC00504, GLCC1, and LINRIS. LINC00504 physically binds to gene promoters, upregulates c-MYC expression, enhances c-myc recruitment to chromatin, and thereby regulates metabolic reprogramming. In CC cells, overexpression of LINC00504 significantly enhanced the expression of many enzymes at the transcriptional level, such as LDHA, HK2, and glyceraldehyde-3-phosphate dehydrogenase (GAPDH), involved in the pentose phosphate, glucose uptake, glycolytic, and tricarboxylic acid (TCA) pathways [39]. These enzymatic changes in turn lead to metabolic outcomes that favor tumor survival. LncRNA GLCC1 stabilizes the ubiquitination of c-Myc transcription factors through direct interaction with the HSP90 chaperon and regulates the expression of LDHA, leading to enhanced glycolysis and CC proliferation and predicting poor clinical prognosis [40]. Yun Wang et al. learned that lncRNA LINRIS is highly expressed in CC and suggests a poor prognosis through clinical specimen analysis, bioinformatics analysis, and CC cell experiments. It blocks the ubiquitination of K139 of IGF2BP2 and the degradation of IGF2BP2 through the autophagy-lysosomal pathway. This stabilizes IGF2BP2 and promotes the downstream effects of IGF2BP2 in turn, such as c-MYC-mediated glycolysis in CC cells [41]. LncRNAs also regulate the activity of pyruvate kinase M2 (PKM2) in CC cells. In CC tissues, downregulation of the long non-coding RNA (lncRNA) HOXB-AS3 results in the elevation of pyruvate kinase M2 (PKM2) levels. This is due to the presence of a conserved 53-amino acid (AA) peptide encoded by HOXB-AS3, which acts against the hnRNPA1-mediated regulation of pyruvate kinase. Consequently, the increased PKM2 expression promotes aerobic glycolysis and leads to glucose metabolic reprogramming [42]. FEZF1-AS1 directly binds to and increases the stability of PKM2 protein, leading to elevated PKM2 levels in the cytoplasm and nucleus, which promotes pyruvate kinase activity and lactate production, further activates STAT3 signaling, and facilitates colorectal cancer development and progression [43]. MAFG-AS1 activates NDUFA4 through binding to miR-147b, leading to the upregulation of pyruvate dehydrogenase kinase isoform 1 (PDK1), phosphofructokinase-1 (PFK1), and PKM2, which ultimately results in the enhancement of invasive capacity and increased lactate production in CRC (colorectal cancer) cells [44]. HNF1A-AS1 regulates MYO6 expression and upregulates HK2 expression in CRC cells via sponge miR-124 to promote cellular glycolysis [45].

Furthermore, lncRNAs reprogram other metabolic processes, including aerobic oxidation, glucose uptake, pyruvate production, and lactate production, in CC cells. LncRNA CRNDE regulates the expression of the metabolic enzyme pyruvate dehydrogenase kinase (PDK) and promotes the expression of GLUTs, thereby inhibiting the aerobic oxidation of glucose and promoting glucose uptake. The expression of lncRNA CRNDE is regulated by insulin/IGFs, and the inhibition of the insulin/IGF-related PI3K/Akt/mTOR or Raf/MAPK pathway can regulate the effect of CRNDE on metabolism in CC cells [46]. Binding directly to miR-216b-5p, the long intergenic non-coding RNA LINC00265 exerts a negative regulatory effect on miR-216b-5p. This, in turn, reduces the inhibition of TRIM44 expression, resulting in the upregulation of glucose metabolism-related enzymes such as glucose transporter (GLUT), pyruvate kinase (PKM), and lactate dehydrogenase (LDH). These changes enhance glucose uptake, pyruvate production, and lactate production, ultimately promoting the proliferation of CC cells. These metabolic alterations are associated with a poor prognosis [47]. In colorectal cancer stem cells (CSCs), the downregulation of lincRNA-p21 leads to the promotion of cancer stemness and tumorigenicity. This occurs through the inhibition of β-catenin expression, which consequently downregulates PDK1. The decrease in PDK1 results in reduced phosphorylation of serine 293 in pyruvate dehydrogenase (PDH), thereby increasing the activity of PDH. Consequently, glucose uptake and lactate production are decreased in cancer cells [48]. LncRNA MALAT1 and Oct4 were highly expressed and miR-20b-5p was lowly expressed in CC cells. Inhibition of lncRNA MALAT1 expression resulted in decreased expression of metabolism-related enzymes GLUT1, HK2, LDH, PKM2, and proteins related to tumor cell stem cell maintenance (Oct4, Nanog, SOX2, and Notch1), and significantly decreased glucose, ATP, and lactate levels in CC cells. The above suggests that LncRNA MALAT1 regulates CC cell metabolism and cell stemness through the miR-20b-5P/Oct4 axis [49]. In colorectal cancer (CC) patients, elevated serum levels of lncRNA AWPPH and GLUT-1 were observed compared to healthy controls. In vitro cellular experiments further confirmed a positive correlation between lncRNA AWPPH and GLUT1. Moreover, it was found that high expression of lncRNA AWPPH promotes GLUT1 expression, leading to increased glucose uptake by intestinal cancer cells. This, in turn, facilitates the proliferation of intestinal cancer cells [50]. In conclusion, lncRNAs regulate the activities of key enzymes in glucose metabolism through different mechanisms, thus affecting the pentose phosphate pathway, glucose uptake, glycolysis, and tricarboxylic acid cycle in CC cells. Upregulation of LncRNA-HCG11 is observed in both colon cancer tissues and cell lines, indicating a poor prognosis. HCG11 promotes the expression of PDK4, a key enzyme for glucose metabolism in CC cells, by binding to miR-144-3p. The above process promotes CC cells’ glucose metabolism, which favors CC cells proliferation, migration and invasion, and increases resistance to 5-FU [51].

### LncRNAs participate in glucose metabolism in gastric cancer (GC)

In GC cells, lncRNAs have been reported to mediate glucose metabolic reprogramming by regulating the rate of glycolysis and glucose uptake. MACC1 has been suggested to be an oncogene in GC with a metabolic role in glycolysis that promotes GC progression. In gastric cancer (GC) tissues, the expression of lncRNA MACC1-AS1, an antisense lncRNA of MACC1, is notably higher compared to paracancerous tissues. Under metabolic stress, lncRNA MACC1-AS1 is induced, leading to the stabilization of MACC1 mRNA and enhanced post-transcriptional MACC1 expression. This upregulation significantly increases the expression of genes associated with glucose uptake and glycolysis (GLUT1, HK2, G6PD, and LDH) at both the mRNA and protein levels. As a result, lncRNA MACC1-AS1 promotes gastric cancer (GC) cell proliferation, inhibits apoptosis, and is correlated with advanced clinical stage and poor prognosis [52]. LncRNA Ftx enhances aerobic glycolysis by up-regulating the expression of the key glycolytic enzyme HK2, which promotes the ability of GC cells to proliferate, invade, and form colonies, and the discovery of this mode of regulation provides promising drug targets to improve the therapeutic strategy of GC [53]. In many types of human cancers, DLX6 antisense RNA 1 (DLX6-AS1) is a lncRNA that exhibits a carcinogenic effect. According to a recent study, DLX6-AS1 is overexpressed in gastric cancer (GC) tissues and cells. The silencing of DLX6-AS1 had a significant impact on reducing glucose uptake, lactate levels, and ATP production ability in GC cells. In addition, shDLX6-AS1-expressing GC cells had significantly lower levels of 3-phosphoinositide-dependent protein kinase 1 (PDK1), a miR-4290 target, compared to cells expressing control shRNA. Furthermore, DLX6AS1 knockdown can also effectively decrease the rate of glycolysis rate and increase mitochondrial oxygen consumption. In conclusion, DLX6-AS1 targets miR-4290 and PDK1 to regulate tumor growth and aerobic glycolysis in GC. Thus, inhibiting DLX6-AS1 can be a promising clinical strategy for treating gastric cancer (GC) [54]. The dysregulation of long non-coding RNA insulin growth factor 2 antisense (IGF2-AS) is associated with tumorigenesis in GC. A recent study reveals that IGF2-AS can act as a miR-195 sponge, and CREB1 is directly targeted by miR-195. Additionally, silencing IGF2-AS relieves the sponging effect of miR-195, leading to elevated CREB1 expression and decreased HK2 expression. This effect results in reduced glucose consumption and lactate production, as well as increased apoptosis in GC cells [55]. A recent study revealed that the expression of lncRNA musculin antisense RNA 1 (MSC-AS1) in GC tissues was considerably higher than that in non-tumor tissues. Next, the study demonstrated that MSC-AS1 knockdown inhibited glucose consumption, pyruvate production, lactate production, and proliferation of GC cells. Conversely, MSC-AS1 overexpression will enhance the glycolytic rate and proliferation of GC cells. The up-regulation of MSC-AS1 in GC cells promotes the mRNA and protein expression of PFKFB3, which enhances cancer cell proliferation, migration, and invasion. However, it does not affect on the expression of HK2 and PKM2 [56]. High expression of LINC00242 is observed in GC tissue samples and cells. In vitro studies demonstrated that silencing LINC00242 significantly slowed down gastric carcinogenesis and progression and inhibited proliferation and glycolysis progression in GC cells. Through screening, it was discovered that miR-1-3p could directly target and bind to LINC00242 and G6PD 3'UTR, respectively. LINC00242 competitively bound miR-1-3p, thus counteracting the inhibition of G6PD by miR-1-3p, suggesting that LINC00242 regulates GC cells proliferation and glycolysis progression through the miR-1-3p/G6PD signaling pathway regulating aerobic glycolysis and proliferation of GC cells [57]. In recent years, the study of lncRNA in GC drug resistance has gradually increased. Elevated expression of both small nucleolar RNA host gene 1 (SNHG1) [58] and lncRNA DLEU2 [59] is observed in GC tissues and cell lines, which indicate a poor prognosis. Functioning as a ceRNA, SNHG1 binds to and suppresses the expression of miR-216b-5p, resulting in an elevation of the downstream regulatory protein HK2 of miR-216b-5p. This subsequently promotes glycolysis and proliferation of GC cells and contributes to the development of resistance to Taxol. Within GC cells, there exists a negative correlation between DLEU2 and miR-30c-5p. This specific miRNA directly targets the 3ʹUTR of LDHA, resulting in the inhibition of its expression. Due to the high expression of DLEU2 in GC cells, it led to a decrease in miR-30c-5p and an increase in LDHA, thus this mechanism facilitates glucose metabolism and enhances Taxol resistance in GC cells.

### LncRNAs participate in glucose metabolism in liver cancer

LncRNAs promote glycolysis and glucose uptake, and reduce aerobic oxidation of liver cancer cells. LncRNA Ftx is upregulated in human hepatocellular carcinoma (HCC) tissues and cell lines and correlates with aggressive clinicopathological features. It was shown that lncRNA Ftx induces PPARγ overexpression to reduce the expression of the downstream signaling proteins PDK1, PFKL, and GLUT4 in HCC cells and promotes cellular aerobic glycolysis and HCC cell progression [60]. LncRNA MEG3 expression was downregulated in human HCC tissues, positively correlated with miR122 and PTEN, and negatively correlated with PKM2. LncRNA MEG3 promotes the expression and maturation of miR122 and inhibits the expression of PKM2 by targeting through miR122, which reduces the expression of liver cancer cells cell-cycle protein D1 and C-Myc, and inhibits the proliferation and differentiation of HCC cells [61]. LncRNA CUDR forms a complex with double mutant P53 (N340Q/L344R), which binds to the PKM2 promoter and enhances PKM2 expression, phosphorylation, and polymer formation, thereby increasing glycolysis and promoting growth of HCC cells [62]. LINC01554 is downregulated in HCC and correlates with tumor size, adjacent organ invasion, advanced tumor stage, and poor prognosis. Within HCC cell lines and clinical tissue samples, miR-365a-3p is found to be up-regulated. This miRNA directly targets the 3ʹ-UTR of LINC01554, thereby causing repression of LINC01554 expression at the transcriptional level. The latter could mediate the ubiquitination of PKM2 and block glycolysis, inhibiting HCC cell proliferation [63]. In HCC patients, the expression of miR675 is upregulated. Through its suppression of the isoforms of heterochromatin 1 (HP1α, HP1β, HP1γ) in human HCC cells, there is a significant decrease in total histone H3 lysine 9 trimethylation (H3K9me3) and histone H3 lysine 27 trimethylation (H3K27me3), as well as an increase in histone H3 lysine 27 acetylation (H3K27Ac). As a result of the decrease in H3K9me3 and H3K27me3, coupled with the increase in H3K27Ac occupancy within the promoter region of EGR1, transcription, sumoylation, and activation of EGR1 are enhanced. Subsequently, there is an up-regulation in the expression of lincRNA H19, which induces and activates the expression of PKM2, ultimately promoting the growth and survival of HCC cells [64]. Both in vitro and in vivo, the proliferation, growth, and metastasis of liver cancer cells can be inhibited by lncRNA WFDC21P. Guan et al. found that a positive correlation was observed between the expression of WFDC21P and Nur77 in clinical HCC samples, which specifically assembled the promoter region of the WFDC21P to promote the expression of WFDC21P, which was significantly inhibited by interacting with both PFKP and PKM2 to inhibit glycolysis, thereby significantly inhibiting the proliferation and metastasis of liver cancer cells [65]. HIF1, which is highly expressed in liver cancer cells, plays a key role in lncRNA-mediated glucose metabolism. HIF1A can bind to the promoter region of lncRNA RAET1K to activate its transcription. LncRNA RAET1K can downregulate the expression of miR-100-5p, leading to an increase in glucose uptake and lactate concentration in liver cancer cells. The progression of HCC is influenced by the regulation of glycolysis in HCC cells induced by hypoxia through the HIF1A/lncRNA RAET1K/miR-100-5p axis [66]. In a recent study, Xin et al. [67] discovered that hypoxia-inducible lncRNA MALAT1 is essential for the enhancement of glycolysis by arsenite. Inhibition of MALAT1 through knockdown suppresses the impact of arsenite on glucose consumption, lactate production, as well as the expression of glycolytic genes including HK-2 and GLUT-4. Conversely, upregulation of MALAT1 disrupts HIF-1α hydroxylation, interferes with HIF-1α-VHL interaction, and leads to HIF-1α stabilization, ultimately promoting glycolysis. These findings suggest that arsenite-induced Warburg effects can be mediated by lncRNAs, which may play a role in arsenite carcinogenesis. A study has shown that upregulation of lncRNA-SOX2OT is observed in HCC and found to induce metastasis by upregulating PKM2. This effect of lncRNA-SOX2OT leads to an increase in the rate of glycolysis in HCC cells, thus facilitating epithelial-mesenchymal transition (EMT). The overexpression of miR-122-5p impedes the lncRNA-SOX2OT-mediated increase in PKM2 expression and the promotion of glucose uptake, glycolysis, and lactate production, ultimately inhibiting HCC cell metastasis [68]. In HCC samples, the expression of lncRNA SLC2A1-AS1 is commonly suppressed. Recent research reveals that SLC2A1-AS1 plays a critical role in inhibiting glycolysis and HCC progression by reducing the mRNA and protein expression of GLUT1, thereby rendering GLUT1 inactive [69]. In hepatocellular carcinoma (HCC), the expression of lncRNA LOXL1-AS1 was found to be significantly higher in both tumor tissues and cells compared to normal liver tissues and cells. Moreover, according to Kaplan–Meier analysis, patients in the high-expression group exhibited a poorer prognosis compared to those in the low-expression group. In liver cancer cells treated with 5 and 20 mM glucose, the inhibition of LOXL1 AS1 resulted in a significant reduction in glucose uptake. This finding suggests that LOXL1 AS1 plays a role in promoting glucose metabolism in liver cancer [70]. Fat mass and obesity-associated (FTO) is a demethylase of N6-methyladenosine (m6A). FTO-IT1, a long non-coding RNA (lncRNA) derived from an intronic region of the FTO gene, functions by recruiting the ILF2/ILF3 protein complex to the 3ʹUTR of FTO mRNA. This interaction leads to the stabilization of FTO mRNA. In HCC cells, high expression of FTO- IT1, which promotes FTO stabilisation, attenuates the m6A modification of glycolysis-related genes PKM2 and GLUT1, which promotes glycolysis in HCC cells and leads to malignant growth [71].

### LncRNAs participate in glucose metabolism in esophageal cancer (EC)

The expression of lncRNA NBAT-1 in EC cells is lower than that in normal esophageal tissues. The low expression of lncRNA NBAT-1 is correlated with the poor prognosis of patients with EC. LncRNA NBAT-1 inhibits cell proliferation and glycolysis in EC by suppressing the expression of PKM2 protein, which is a key metabolic enzyme of glycolysis [72]. Ma et al. [73] found that the expression of HK2 was suppressed by miR-125 and miR-143. However, the lncRNA HOTAIR acted as a competing endogenous RNA, sponging miR-125 and miR-143, consequently promoting HK2 expression. Through this process, lncRNA HOTAIR could facilitate the glycolysis and tumorigenesis of esophageal squamous cell carcinoma (ESCC) cells in vitro and in vivo. AGPG, a long non-coding RNA, prevents the proteasomal degradation of PFKFB3 by inhibiting its ubiquitination mediated by APC/C, which leads to the accumulation of PFKFB3 in EC cells, thereby activating cellular glycolysis and promoting their proliferation [74]. LINC00184 regulates the activities of key glycolytic enzymes HK2, LDHA, and PDH, a key enzyme of mitochondrial oxidative phosphorylation. It achieves this by enhancing the methylation of the PTEN promoter, thus inhibiting PTEN expression and promoting Akt phosphorylation. The above processes promote glycolysis and mitochondrial oxidative phosphorylation in EC cells, thereby promoting the proliferation, migration, and growth of EC cells [75]. LncRNA SLC2A1-AS1 induced by GLI3 can sponge miR-378a-3p to enhance GLUT1 expression leading to increased glycolysis and EMT progression, all of which promote esophageal squamous cell carcinoma migration, invasion, and growth, and suppress apoptosis in vitro and in vivo [76]. LncRNA G077640 is up-regulated in human ESCC tissues and cells, which clinically suggests a poor prognosis for patients. G077640 can directly interact with histone H2AX to block HIF1α degradation and promote the expression of glucose metabolism enzymes, such as GLUT4, HK2, and PDK1, thus promoting the proliferation and migration of ESCC cells [77].

### LncRNAs participate in glucose metabolism in pancreatic and gallbladder cancers

Qi et al. found that lncRNA MACC1-AS1 expression is upregulated in pancreatic cancer (PC) tissues and cells and represents a poor prognosis. In PC cells with high expression of MACC-AS1, there was a corresponding increase in PAX8 protein and pyruvate kinase, and increased NOTCH1 phosphorylation, which promoted PC glycolysis and progression. Inhibition of MACC1-AS1 resulted in reduced proliferation and metastasis of PC cells [78]. By analyzing clinical specimens of PC tissues, Yu et al. found that the levels of lncRNA HOTAIR and HK2 were higher than those of adjacent healthy tissues, and that overexpression of lncRNA HOTAIR and HK2 in PC cells promoted their proliferation. Overexpressing the long non-coding RNA HOTAIR significantly increased HK2 protein levels, as well as the production of lactate, uptake of glucose, and ATP production in PC cells [79]. In PC, aberrant expression of LINC01448 was found to reduce miR-505 expression, resulting in the inhibition of cell proliferation, invasion, sphere formation, glucose consumption, and lactate production. This was achieved by targeting HK2, according to a recent study. Regulating PC glycolysis and progression is critically influenced by the LINC01448/miR-505/HK2 pathway, implying its significant role, as suggested by recent findings [80]. LncRNA PVT1 is an important epigenetic regulator that plays an important role in tumorigenesis. LncRNA PVT1 was found to be highly expressed in pancreatic ductal adenocarcinoma tissues and reduced patient survival. Sun et al. hypothesized that lncRNA PVT1 binds to and inhibits miR- 519d-3p, thereby suppressing HIF-1 expression. Down-regulation of PVT1 in PC cells reduced the expression of GLUT1, HK2, and LDHA, thereby inhibiting glycolysis in PC cells. The above processes have been shown to promote glycolysis, proliferation, and invasion of pancreatic ductal adenocarcinoma cells [81]. By analyzing pancreatic tumor tissues and bioinformatics analysis, hu et al. found that the lncRNA DICER1-AS1 is lowly expressed in PCs, in contrast to the expression of some glycolytic genes. Mechanistically, to promote DICER1 transcription, the lncRNA DICER1-AS1 recruits the transcription factor YY1 to the DICER1 promoter, according to recent studies. Meanwhile, DICER1 can promote the maturation of miR-5586-5P, which inhibits the expression of glycolytic genes LDHA and HK2, and suppresses glycolysis, proliferation, and metastasis of PC cells [82]. Abnormal expression of PVT1 has been reported in gallbladder cancer. Chen et al. [83] showed that PVT1 upregulates HK2 expression through its competitive endogenous RNA (ceRNA) activity against miR-143 in gallbladder cancer cells. Moreover, the PVT1/miR-143/HK2 axis promotes cell growth and metastasis by modulating aerobic glucose metabolism in gallbladder cancer cells.

## LncRNAs modulate lipid metabolism in GI tract tumors through metabolic reprogramming

Abnormal lipid metabolism is another feature of tumor metabolic reprogramming, which is characterized by changes in lipid uptake, storage, lipogenesis, and decomposition [84]. Tumor cells require a higher amount of lipids than normal cells. Lipids are required as raw materials for cell membrane synthesis, biomolecule synthesis, signal transduction of cancer cells, and inflammation and vascular regulation, which lead to the upregulation of pathways involved in FA synthesis in tumor cells. Tumor cells are characterized by altered expression of key enzymes required for lipid metabolism. Acetyl-CoA carboxylase (ACC) is a key enzyme in FA synthesis. In the occurrence and development of tumors, the metabolism of fatty acids mediated by ACC (acetyl-CoA carboxylase) plays a significant role, as studies have shown [85]. The expression of fatty acid synthase (FASN), a key enzyme in the last step of catalytic FA synthesis, is significantly higher in a variety of human tumor cells than in corresponding normal cells. Moreover, in tumor growth and survival, FASN (fatty acid synthase) plays a crucial role, according to recent evidence [86]. Stearoyl CoA desaturase 1 (SCD1) is an essential lipogenic enzyme responsible for synthesizing two monounsaturated fatty acids, oleate and palmitoleate, which are integral components of cell membrane lipids [87]. The requirement of these FAs as raw materials is higher in tumor cells than in normal cells, which may explain the high expression of SCD in tumor cells. Moreover, the production of unsaturated FAs caused by increased SCD activity enhances tumor cell resistance [88]. Moreover, lipids not only serve as raw materials but also provide energy to tumor cells. Cancer cells can also catabolize fat to meet their bioenergetic needs. Fatty acids are mainly metabolized by oxidative decomposition. As a key enzyme in the first step of fatty acid oxidation, acyl CoA synthase (ACS) is highly expressed in CC and is related to patient prognosis [89]. Fat mobilization is the first step of catabolism, in which adipose triglyceride lipase (ATGL) is an important lipase. Adipose mobilization can be activated and fatty acid oxidation can be promoted in tumors, which can also facilitate tumor development [90]. Therefore, the energy produced by the oxidative decomposition of fatty acids drives tumor cell growth. Realizing the importance of lipid metabolism in tumor cells, researchers have explored the mechanisms underlying lipid metabolic reprogramming in tumors. Various molecules, pathways, and genes related to lipid metabolism have been discovered in recent years. Next, we will explain how lncRNAs affect metabolic reprogramming through the abovementioned key enzymes of lipid metabolism in the following four types of cancer: CC, liver cancer, intrahepatic cholangiocarcinoma (ICC), and PC (Fig. 2 and Table 2).

**Table 2:** LncRNAs involved in the regulation of lipid metabolism of gastrointestinal cancer cells

Tumor type	LncRNA	Expression	Metabolism-related enzyme	Metabolic process	Clinical prognosis	Ref
Colorectal cancer	LINC00504	Up	FASN	Lipid synthesis	–	[39]
	CRNDE	Up	FASN	Lipid synthesis	–	[46]
	SNHG16	Up	SCD	Lipid synthesis	–	[91]
Liver cancer	HULC	Up	ACSL1	Lipid synthesis	–	[92]
	NEAT1	Up	ATGL	Lipid synthesis	Poor	[93]
	RP11-386G11.10	Up	FASN, ACC, SCD	Lipid synthesis, lipolysis	Poor	[94]
Cholangiocarcinoma	HAGLROS	Up	FASN, ACC, SCD1	Lipid synthesis, lipolysis	Poor	[95]
Gastric cancer	MACC1-AS1	Up	CPT, ACS	Lipolysis	Poor	[97]
	PEP-AKR1C2	Up	CPT	Lipolysis	Poor	[98]
Pancreatic cancer	NONHSAT105177	Down	HMGCR	Lipid synthesis	–	[96]

*FASN* Fatty acid synthase, *ACC* Acetyl-CoA carboxylase, *ATGL* Adipose triglyceride lipase, *SCD* Stearoyl CoA desaturase, *ACS* acetyl-CoA synthase, *CPT* Carnitine palmitoyl transferase, *ACSL1* ACS long-chain family member 1, *HMGCR* Hydroxy methylglutaryl coenzyme CoA reductase

### LncRNAs participate in lipid metabolism in colon cancer (CC)

Several lncRNAs, such as LINC00504 and CRNDE, have been reported to be involved not only in glucose metabolism but also in lipid metabolism. In CC cells, the significant transcriptional regulator of c-Myc is known as LINC00504. When LINC00504 is overexpressed in CC cells, the level of FASN is correspondingly elevated, which promotes the production of citric acid and thus fatty acid synthesis. In addition, LINC00504 accelerated tumor progression in CC cells via the c-Myc/FASN axis [39]. The lncRNA CRNDE is highly expressed in CC cells and upregulates the expression of FASN, which induces abnormal lipid metabolism and promotes the progression of CC. LncRNA CRNDE is regulated by insulin/IGF, and the inhibition of the insulin/IGF-related PI3K/Akt/mTOR pathway or Raf/MAPK pathway can regulate the effect of CRNDE on cell metabolism, thereby serving as an effective therapeutic strategy against CC [46]. LncRNA SNHG16 is highly expressed in CC tissues and cells, where it acts as a ceRNA that binds to the 3'UTR of its target miRNA, stearoyl coenzyme desaturase (SCD). Silencing SNHG16 in CC cells can down-regulate the expression of SCD, thereby inhibiting lipid synthesis and metabolism in CC cells, reducing the cells’ viability, inducing apoptotic death, and decreasing migration [91].

### LncRNAs participate in lipid metabolism in liver cancer

LncRNA HULC is up-regulated in HCC tissues and cells, which promotes methylation of CpG islands in the promoter of miR-9 and inhibits miR-9 expression. MiR-9 is able to target the 3ʹ UTR of the transcription factor PPARA, which activates the promoter of the acyl-CoA synthase subunit (ACSL1). The up-regulation of PPARA, caused by a decrease in MiR-9, leads to elevated expression of ACSL1. This increase in ACSL1 expression promotes adipogenesis, enhances intracellular accumulation of triglycerides and cholesterol, and ultimately stimulates the growth of hepatocellular carcinoma cells. Through a positive feedback loop involving the retinoid receptor RXRA, cholesterol accumulation activates the HULC promoter, resulting in an upregulation of HULC expression [92]. Furthermore, high expression of triglyceride lipase (ATGL) in human liver cancer tissues leads to high levels of diacylglycerol (DAG) and free fatty acid (FFA) and is associated with a poor prognosis. Liu et al. found that lncRNA NEAT1 up-regulated the expression of ATGL through miR-124-3p binding, which increased the levels of DAG and FFA, and promoted the growth of HCC cells. NEAT1 modulates the survival of HCC cells through the miR-124-3p/ATGL/DAG+FFA pathway, which may be an important target for the treatment of HCC [93]. LncRNA RP11-386G11.10 is overexpressed in HCC, suggesting a poor prognosis.RP11-386G11.10 can act as a ceRNA that competitively binds to miR-345-3p and promotes the expression of the nuclear protein HNRNPU and downstream lipid metabolism-associated enzymes, FASN, ACC, and SCD, which leads to lipid accumulation in HCC cells and promotes HCC cells proliferation and metastasis [94].

### LncRNAs participate in lipid metabolism in other GI tract tumors

LncRNAs have been reported to participate in lipid metabolism in several other GI tract tumors. LncRNA HAGLROS plays an important role in lipid metabolism in ICC cells, upregulated HAGLROS expression leads to the increase in the activation of lipid-related proteins such as FAS, ACC, SCD1, and Carnitine palmitoyl transferase 1 (CPT1) in tumor cells, indicating a poor prognosis. A study showed that the silencing of HAGLROS regulated the mTOR signaling pathway to inhibit the activation of lipid-related proteins [95]. In pancreatic ductal carcinoma (PDAC) tissues, the expression of lncRNA NONHSAT105177 is noticeably diminished compared to the adjacent non-cancer tissues. In PDAC cells, NONHSAT105177 overexpression significantly down-regulated the expression of cholesterol pathway genes HMGCR, CLU, and LSS, thereby reducing cholesterol biosynthesis and inhibiting PDAC cell growth [96]. LncRNA MACC1-AS1, a glucose-regulating lncRNA, is also reported to be involved in lipid metabolism. In GC cells, mesenchymal stem cells (MSCs) secrete TGF-β1 and lead to the up-regulation of MACC1-AS1 through the activation of Smad2/3. LncRNA MACC1-AS1 binds directly to miR-145-5p and promotes the expression of the key fatty acid oxidases, CPT and ACS [97]. Exo-lncAKR1C2 is a gastric cancer cell-secreted lncRNA that encodes a micro-protein, PEP-AKR1C2, which reduces the phosphorylation level of YAP and promotes the expression of CPT1A, thereby promoting fatty acid oxidation and ATP production in lymphatic endothelial cells. The above process promotes tube formation and migration of lymphatic endothelial cells, lymphangiogenesis and lymphatic metastasis, leading to gastric cancer progression and poor prognosis. This process enhances the fatty acid oxidation-dependent stemness and chemoresistance of GC cells [98].

## LncRNAs participate in amino acid metabolism in GI tract tumors through metabolic reprogramming

Hans Krebs proposed the famous TCA cycle in 1937, pointing out the important role of the TCA cycle in providing cellular energy. Later, researchers found that when pyruvate supply was insufficient to affect the TCA cycle, glutamine could generate oxaloacetic acid and acetyl-CoA through catabolism. This phenomenon was recognized as an alternative to the TCA cycle [99]. Glutamine has been found to be crucial for both normal and cancer cell growth, as subsequent studies have shown that the consumption of glutamine can provide the necessary energy and biomolecules for tumor cell proliferation [100]. In normal cells, glutamine is catalyzed by glutaminase (GLS) to glutamate, which is then catalyzed by glutamate dehydrogenase (GDH) to α-ketoglutarate, eventually entering the TCA cycle [101]. In tumor cells, glutamine metabolism becomes overactive. While the Warburg effect of tumor cells leads to the downregulation of the TCA cycle, glutamine metabolism serves as an “anaplerotic reaction” for the TCA cycle [12]. In brief, glutamine metabolism arises as a result of tumor metabolic reprogramming. In addition to abnormal glutamine metabolism, changes in serine/glycine metabolism in tumor cells may contribute to amino acid metabolic reprogramming [102]. In tumor cells, the enhanced expression of serine hydroxymethyltransferase 2 (SHMT2) leads to the excessive catalysis of serine into glycine [103], which participates in the tetrahydrofolic acid reaction, promotes the metabolism of one carbon unit, and synthesizes the nucleotides, lipids, and proteins required for cell proliferation [104]. LncRNAs play an essential role in glutamine metabolic reprogramming and serine/glycine metabolism in GI tract tumor cells, mainly by acting through the glutamine metabolism-related key enzymes GLS and GDH and the serine/glycine metabolism-related key enzyme SHMT2 (Fig. 3 and Table 3). Reprogramming of amino acid metabolism has also been reported in tumors of the GI tract, such as PC, ICC, and CC.

**Table 3:** LncRNAs involved in the regulation of amino acids metabolism of gastrointestinal cancer cells

Tumor type	LncRNA	Expression in tumors	Metabolism-related enzyme	Metabolic process	Clinical prognosis	Ref
Pancreatic cancer	GLS-AS	Down	GLS	Glutamine	Poor	[105]
	XLOC_006390	Up	GDH	Glutamine	Poor	[106]
Liver cancer	TUG1	Up	GDH	Glutamine	Poor	[107]
Colorectal cancer	LINC01234	Up	SHMT2	Serine/glycine	Poor	[108]

*GLS* Glutaminase, *GDH* Glutamate dehydrogenase, *SHMT2* Serine hydroxymethyltransferase 2

### LncRNAs participate in amino acid metabolism in PC

LncRNAs interact with c-Myc to regulate glutamine metabolism in PC cells. The expression of the antisense lncRNA of glutaminase (GLS-AS) is lower in PC than that of non-tumor adjacent tissues. At the post-transcriptional level, GLS-AS induces a conformational change in glutaminase (GLS) pre-mRNA, leading to the formation of double-stranded RNA. This, in turn, impairs GLS expression through an ADAR/Dicer-dependent RNA interference mechanism. Moreover, in PC cells, a reciprocal feedback loop has been reported between Myc and GLS-AS, which controls the overexpression of GLS. The proliferation and invasion of PC cells were inhibited by ectopic overexpression of GLS-AS through inhibition of the Myc/GLS pathway [105]. In PC, lncRNA XLOC_006390 functions by inhibiting the ubiquitination of c-Myc, thereby enhancing the protein stability of c-Myc. It has been shown that c-Myc binds to the promoter of glutamate dehydrogenase 1 (GDH1) and positively regulates the expression of the GDH1 gene, thereby catalyzing the transformation of glutamic acid into α-ketoglutarate and promoting PC progression [106].

### LncRNAs participate in amino acid metabolism of other GI tract tumors

The participation of lncRNAs in amino acid metabolism has been reported in other GI tract tumors, including ICC and CC. The overexpression of lncRNA taurine-upregulated gene 1 (TUG1) can predict a poor prognosis in patients with ICC. Zeng et al. [107] revealed that TUG1, functioning as a ceRNA, binds to miR-145, thus repressing the decomposition of Sirt3 mRNA and enhancing the expression of Sirt3 and GDH proteins. In contrast, reducing TUG1 expression in ICC cells resulted in a significant decrease in glutamine consumption, α-ketoglutarate production, and ATP levels, while inhibition of miR-145 reversed these effects. The results suggest that the TUG1/miR-145/Sirt3/GDH pathway may provide a new therapeutic target for ICC treatment. Lin et al. [108] found that overexpression of LINC01234 in CC tissues led to significant upregulation of serine hydroxy methyl transferase 2 (SHMT2), which was associated with a poor prognosis. SHMT2 is a target of miR-642a-5p. By functioning as a competing endogenous RNA (ceRNA), LINC01234 can sponge miR-642a-5p, thereby attenuating its inhibitory effect and promoting the expression of SHMT2. Therefore, glycine-mediated metabolism is associated with cell proliferation and the progression of CC.

## Role of lncRNAs in the tumor microenvironment (TME) and immune regulation

**Fig. 4:**
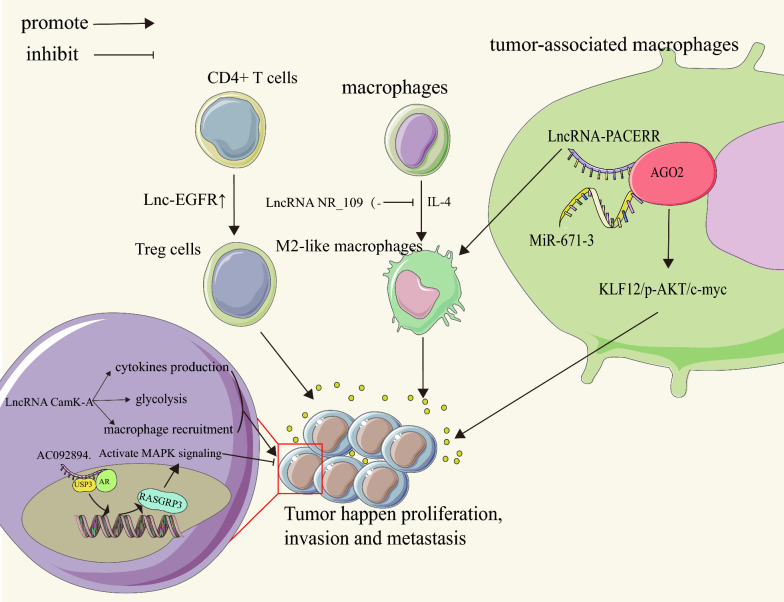
Role of lncRNAs in the tumor microenvironment and immune regulation

The tumor immune microenvironment (TME) comprises infiltrating immune cells, as well as inflammatory cytokines or chemokines and is a defining feature of tumors. This immune microenvironment plays a crucial role in both inhibiting tumor growth through an anti-tumor immune response and promoting tumor growth through tumor immune escape. LncRNAs can regulate the development and differentiation of tumor-associated immune cells, thereby influencing the body’s anti-tumor immune response. Simultaneously, lncRNAs also modulate the expression of immune-related cytokines and associated pathways by influencing immune cell function, consequently impacting the tumor-related immune microenvironment (Fig. 4).

Within the TME, Tregs are responsible for inhibiting tumor immunity. Recently, researchers identified a lncRNA called lnc-EGFR that is highly expressed in Tregs. This was discovered via high-throughput sequencing of the lncRNA transcriptome in tumor-infiltrating lymphocytes and peripheral blood lymphocytes of patients with HCC. The upregulation of lnc-EGFR promotes the differentiation of CD4+ T cells into Tregs, which ultimately suppresses the tumor-killing ability of cytotoxic T cells. This promotes the progression of HCC [109]. The important lncRNA molecule, CamK-A, plays a critical role in regulating tumor Ca2+ signaling. It also has a synergistic effect by promoting both tumor metabolic reprogramming and microenvironment remodeling. CamK-A helps cells adapt to hypoxic conditions by enhancing tumor cytokine production, increasing tumor cell glucose uptake, and facilitating macrophage infiltration. These biological changes lead to the abnormal upregulation of tumor glucose metabolism, angiogenesis, and remodeling of the microenvironment, ultimately promoting tumor progression. The study provides a new perspective on how the hypoxic microenvironment impacts tumor metabolic reprogramming and microenvironment remodeling [110].

As crucial components of the TME, tumor-associated macrophages (TAMs) play a significant role in tumor progression. The highly expressed lncRNA NR_109 is predominantly found in M2-like macrophages. In vitro and in vivo studies show that when NR_109 is knocked down, it inhibits the IL-4-induced polarization of M2-like macrophages. This leads to significantly reduced activity in the promotion of proliferation and metastasis of gastric cancer tumor cells [111]. In the TME, the majority of TAMs differentiate into the M2 phenotype, which actively promotes tumor growth and metastasis. In TAMs, there is an observed high expression of lncRNA-PACERR, which is associated with a poor prognosis in patients diagnosed with pancreatic ductal adenocarcinoma (PDAC). The findings of this study provide evidence that lncRNA-PACERR promotes the in vitro and in vivo proliferation, invasion, and migration of tumor cells. It achieves this by increasing the number of M2 polarized cells and activating the KLF12/p-AKT/c-myc pathway through its binding to miR-671-3p [112].

Within the TME, lncRNAs have a crucial role in mediating interactions between tumor and non-tumor cells, as well as other non-cellular components. T In tumor biology, these lncRNAs play a facilitative role in diverse processes, including tumor growth, metastasis, angiogenesis, drug resistance, cellular metabolic reprogramming, and induction of immunosuppression. Additionally, lncRNAs present in the TME hold promise as potential molecular markers for tumor detection or as targets for tumor treatment [113].

Tumor chemotherapy failure can be attributed to chemotherapy drug resistance, which occurs when tumor cells decrease their sensitivity to drugs, thereby reducing drug efficacy or rendering them ineffective. This phenomenon of reduced drug sensitivity in tumor cells is a critical factor contributing to the failure of tumor chemotherapy. In the treatment of colorectal cancer, oxaliplatin resistance stands as a complex process and a significant hindrance. Through its role as a scaffold molecule, AC092894.1 mediates the deubiquitination of AR via USP3. This process subsequently enhances the transcription of RASGRP3. The sustained activation of the MAPK signaling pathway ultimately induces apoptosis in colorectal cancer (CRC) cells [114]. The elevated expression of a recently identified lncRNA, CACClnc, in CRC, is associated with chemoresistance and an unfavorable prognosis. CACClnc facilitates resistance to chemotherapy in CRC by bolstering DNA repair and augmenting homologous recombination, both in vitro and in vivo [115].

Currently, immune checkpoint inhibitors (ICIs) are considered the most promising treatment modality in the field of immunotherapy, offering great potential for overcoming tumors [116]. PD-1/PD-L1 inhibitors have made considerable clinical advancements in the treatment of cancer, specifically for MSI-H/dMMR CC, HCC, and gastric cancer. This progress has marked a significant breakthrough for immune checkpoint inhibitors [117]. LncRNAMIR17HG is responsible for the upregulation of PD-L1 in colorectal cancer. The decrease in the expression of the tumor suppressor B-cell linker (BLNK) caused by miR-17-5p transcribed by MIR17HG enhances the migration and invasion of cancer cells [118]. Currently, to target lncRNAs, various strategies are available, such as small molecule inhibitors, antisense oligonucleotides (ASOs), RNA interference technology, and CRISPR/Cas9 genome editing [119].

## Discussion

Conventional treatments for GI tract tumors include surgical resection, radiotherapy, and adjuvant chemotherapy. Detecting and treating gastrointestinal (GI) tract tumors at an earlier stage can significantly enhance the survival rate of patients and alleviate their burden. As mentioned above, the poor prognosis of GI tract tumors is often linked to aberrant expression of lncRNAs, which have a direct or indirect impact on the metabolic reprogramming of tumor cells and the tumor microenvironment. Therefore, lncRNAs have shown great potential as prognostic biomarkers and therapeutic targets in the diagnosis, prognosis and treatment of GI tract tumor patients.

This review primarily examines the regulatory mechanisms employed by lncRNAs to modulate metabolic reprogramming in GI tract tumor cells, with a special focus on the review of lncRNA-mediated regulation of glucose, lipid, and amino acid metabolism in GI tract tumors. In glucose metabolism, lncRNA regulates key metabolic enzymes such as HK2, PKM, LDHA, and GLUT to affect glucose uptake, aerobic oxidation, pentose phosphate pathway, and other glucose metabolic activities in tumors of the GI tract. The regulatory effect of lncRNA on glucose metabolic reprogramming provides sufficient energy for tumor cells and promotes the rapid growth of tumors. For lipid metabolism, key enzymes such as ACC, FASN, SCD, and ACS are affected by lncRNAs, resulting in lipid uptake, storage, and catabolic reprogramming, through which cancer cells obtain important raw materials for tumor growth and progression. For amino acid metabolism, lncRNAs affect the metabolic reprogramming of amino acids such as serine and glycine through GLS, GDH, SHMT2, and other related key enzymes, thus affecting the progression of tumors. By elaborating on the regulatory processes and mechanisms of lncRNAs in these metabolic pathways, we hope to identify more effective and accurate targets for the treatment of GI tract tumors. A greater knowledge of the biology of metabolic reprogramming will lay the groundwork for employing diet and medications to interfere in metabolism and regulate tumor growth via glucose, fat, and amino acid metabolism, paving the way for advancements in cancer treatment and prevention.

Throughout the progression of tumors, tumor cells continuously interact with their environment. By releasing signaling molecules, such as lncRNAs, tumor cells can impact their microenvironment by fostering the proliferation and migration of neighboring non-tumor cells, inducing immunosuppression, and promoting angiogenesis, among other effects. Aside from lncRNAs, other constituents of the TME can also influence the proliferation and migration of tumor or non-tumor cells, as well as drug resistance. These TME components can impact numerous processes, including tumor progression, through lncRNA-mediated mechanisms.

The altered metabolism of tumor cells leads to significant effects on the tumor microenvironment (TME). These effects include changes in metabolites and pH levels in the microenvironment and alterations in immune cells and the cytokines they secrete, which contribute to the immune escape of tumor cells.

When the tumor microenvironment (TME) encounters conditions like severe nutrient deficiencies and hypoxia, metabolic reprogramming is initiated in tumor cells, immune cells, and stromal cells [120, 121]. While metabolic changes such as glycolysis are selected by tumor cells to support their biosynthesis and energy requirements for rapid proliferation and survival in the hypoxic TME, it has been shown that immune cells (e.g., TAM, T cells, TADC, MDSC, neutrophils, B cells, and NK cells) in the TME also undergo metabolic alterations to promote or inhibit tumor growth. As a significant metabolite of glycolysis, lactate serves as a regulator that connects tumor immunity and tumor growth. The elevated levels of lactate and reduced pH in the microenvironment induce changes in the phenotype and function of immune cells within TME. These changes ultimately shift the development of the immune system towards immunosuppression and immunotolerance [122].

The composition of various cells that exert their influence upon tumor cells results in a complex and varied tumor immune microenvironment. The composition of the immune microenvironment varies from one type of tumor to another. To achieve effective targeted metabolic therapy, it is essential to identify synergistic targets within both tumor cells and immune cells, thereby generating synergistic effects. Despite potential future advancements, targeting specific metabolic enzymes and addressing the adaptability of tumor metabolism remains a significant obstacle. However, the ultimate objective should be a dedication to targeting tumor metabolism and concurrently augmenting anti-tumor immunity, as this is the most desirable outcome.

Despite the immense potential of lncRNAs as therapeutic targets, several challenges need to be addressed in terms of safety and targeting difficulties. However, with ongoing progress and optimization of research, the development of lncRNA-targeted therapy continues to hold vast application prospects for tumor immunity and metabolism. In summary, although there are still various issues that must be resolved, the future of this novel regimen looks promising.

## Data Availability

Not applicable.

## Abbreviations

lncRNAs: Long non-coding RNAs
GI: Gastrointestinal
FA: Fatty acid
FFA: Free fatty acid
GLUT: Glucose transporter
HK2: Hexokinase 2
PK: Pyruvate kinase
LDH: Lactate dehydrogenase
PDK: Pyruvate dehydrogenase kinase
PDK1: Pyruvate dehydrogenase kinase isozyme 1
PFK: Phosphofructokinase
PKM2: Pyruvate kinase type M2
LDHA: Lactate dehydrogenase A
GAPDH: Glyceraldehyde-3-phosphatedehydrogenase
TCA: Tricarboxylic acid
G6PD: Glucose-6-phosphatedehydrogenease
PFKFB3: 6-Phosphatefructose-2-kinase
R5P: 5-Phosphateribose
CC: Colon cancer
TCA: Tricarboxylic acid
GC: Gastric cancer
DLX6-AS1: DLX6 antisense RNA 1
IGF2-AS: Insulin growthfactor2antisense
CREB1: CAMP-responsiveelementbindingprotein1
MSC-AS1: MusculinantisenseRNA1
HCC: Hepatocellular carcinoma
EMT: Epithelial-mesenchymal transition
ESCC: Esophageal squamous cell carcinoma
PDH: Pyruvate dehydrogenase
PC: Pancreatic cancer
ceRNA: Competitive endogenous RNA
FASN: Fatty acid synthase
ACC: Acetyl-CoA carboxylase
ATGL: Adipose triglyceride lipase
SCD: Stearoyl CoA desaturase
ACS: Acetyl-CoA synthase
CPT: Carnitine palmitoyl transferase
ACSL1: ACS long-chain family member 1
HMGCR: 3-Hydroxy-3-methylglutaryl-Coenzyme A reductase
ICC: Intrahepatic cholangiocarcinoma
DAG: Diacylglycerol
TGF-β1: Transforming growth factor β1
MSCs: Mesenchymal stem cells
GLS: Glutaminase
GDH: Glutamate dehydrogenase
SHMT2: Serine hydroxymethyltransferase 2
TUG1: Taurine-upregulatedge
TME: Tumor microenvironment
